# Supplementary material for: An improved auxin-inducible degron system preserves native protein levels and enables rapid and specific protein depletion
Source: Genes Dev. 2019 Oct 1;33(19-20):1441–55. doi: 10.1101/gad.328237.119 (PMC6771385; doi:10.1101/gad.328237.119)
Supplement: Supplemental Material [file supp_33_19-20_1441__index.html]

An improved auxin-inducible degron system preserves native protein levels and enables rapid and specific protein depletion — Supplemental Material 

# An improved auxin-inducible degron system preserves native protein levels and enables rapid and specific protein depletion

## Supplemental Material

- Supplemental\_Tables.docx
- Supplemental\_Legends.docx
- SupplementalFigureS9.pdf
- SupplementalFigureS6.pdf
- SupplementalFigureS10.pdf
- SupplementalFigureS7.pdf
- SupplementalFigureS11.pdf
- SupplementalFigureS8.pdf
- Supplemental\_Material.docx
- SupplementalFigureS3.pdf
- SupplementalFigureS1.pdf
- SupplementalFigureS4.pdf
- SupplementalFigureS2.pdf
- SupplementalFigureS5.pdf
- SupplementalFigureS12.pdf
