# Supplementary material for: An improved auxin-inducible degron system preserves native protein levels and enables rapid and specific protein depletion
Source: Genes Dev. 2019 Oct 1;33(19-20):1441–55. doi: 10.1101/gad.328237.119 (PMC6771385; doi:10.1101/gad.328237.119)
Supplement: Supplemental Material [file supp_gad.328237.119_Supplemental_Tables.docx]

| **Name** | **Sequence** |
| --- | --- |
| ZNF143F | A*A*GAAGCCATCAGAATAGCGTCTAGAATCCAACAAGGAGAAACGCCAGGGCTTGACGACGGTGGATCTGGAGGTTCAGGTGGCAGTGTCGAGCTGAATCT |
| ZNF143R | A*A*GACTCCTTCTGCTTTATTGCTCCATTGTTCTGAGGATTAATCATCCAATCAGTTAGCCTCCCCCATCTC |
| TEAD4F | T*G*TCTTTGAGGTGTCAGCCAGTGAGCACGGGGCTCAGCACCACATCTACAGATTAGTTAAGGAGGGTGGATCTGGAGGTGGCAGTGTCGAGCTGAATCT |
| TEAD4R | C*G*TCTCTTCCCCCCTCCCTGCTCCCCGAGTCTCTCATTCTTTCACCAGCCTCAGTTAGCCTCCCCCATCTC |
| p53F | A*G*TCTACCTCCCGCCATAAAAAACTCATGTTCAAGACAGAAGGGCCTGACTCTGATGGTGGATCTGGAGGTTCAGGTGGCAGTGTCGAGCTGAATCT |
| p53R | G*G*GGTGGGAGGCTGTCAGTGGGGAACAAGAAGTGGAGAATGTCAGTCTGATCAGTTAGCCTCCCCCATCTC |

## Supplemental Material

**Supplemental Table S1**

**PCR Homology Donor Construct Primers**

**Supplemental Table S2**

| **Name** | **Sequence** |
| --- | --- |
| TEAD4 | TCAGCACCACATCTACAGGC |
| ZNF143 | GAGGATTAATCATCCAACCC |
| p53 | GGAGAATGTCAGTCTGAGTC |

**sgRNA Primers (DNA target sequence)**
