# Supplementary material for: An improved auxin-inducible degron system preserves native protein levels and enables rapid and specific protein depletion
Source: Genes Dev. 2019 Oct 1;33(19-20):1441–55. doi: 10.1101/gad.328237.119 (PMC6771385; doi:10.1101/gad.328237.119)
Supplement: Supplemental Material [file supp_gad.328237.119_Supplemental_Legends.docx]

**Supplemental Fig. legends**

**Supplemental Fig. S1.** ARF is critical for the proper regulation of the auxin-induced transcriptional response in plants. In the absence of auxin, IAA binds the PB1 domain of ARF. Auxin binds to TIR1 and drives strong association of TIR1 with IAA to mediate ubiquitination and degradation.

**Supplemental Fig. S2.** TIR1 was integrated into the AAVS1 locus of HEK293T cells. A) A schematic of the strategy to integrate CMV-driven TIR1 into the AAVS1 locus of the HEK293T cells using the CRISPR-Cas9 system. Primers F and R2 generate an amplicon if the construct is inserted. F and R primers flank the insert and will amplify if the construct does not insert into a copy of AAVS1. B) At least one copy of AAVS1 contains the insertion in both clone 4 and clone 8. C) All copies of AAVS1 contain the insertion in clone 4, but clone 8 is heterozygous. D) Western blotting indicates that the TIR1 protein is expressed in both clones.

**Supplemental Fig. S3.** Chronic auxin-independent depletion is proteasome dependent. A) Two independent clones (1 and 2) of ZNF143-AID tagged cells were treated with MG132 for four and a half hours. ZNF143 protein levels increase after MG132 treatment. B&C) TIR1 depletion by siRNA knockdown stabilizes ZNF143-AID and TEAD4-AID. D) An auxin-independent interaction between ZNF143-AID and TIR1 is proposed to drive chronic proteasomal degradation.

**Supplemental Fig. S4.** Expression of the ARF domains rescues chronic protein depletion, but does not detectably affect protein levels in the absence of AID-tagging. A) A schematic of the strategy to express ARF (PB1 domain of ARF16 or the MR and PB1 domain of ARF25) within HEK293T cells that express TIR1 and AID-tagged TEAD4. B) TEAD4-AID protein is stabilized after expression of GFP-ARF16-PB1 and GFP-ARF25-MR-PB1, but not stabilized by the empty vector that expresses GFP. C) The illustrated workflow highlights the source of each lane in Figure 2B. D) Expression of the ARF16-PB1 domain rescues ZNF143 levels to 51% to 64% the level of untagged ZNF143. E) ARF16-PB1 and TIR1 expression had no effect on the expression of ZNF143, TEAD4, and p53; actin and phosphorylated H3S10 are loading controls.

**Supplemental Fig. S5.** ARF rescue increases the rate of auxin dependent degradation of AID tagged protein. HEK293T-TIR1 ZNF143-AID cells (A) and ARF-16-rescued cells (B) were treated with cycloheximide alone, or cycloheximide in combination with auxin, to compare degradation kinetics upon inhibiting translation. C) Densitometric quantification measurements of ZNF143-AID bands from the lower blots in panel A (green trace) and panel B (red trace) were plotted and fit to a one phase decay equation.

**Supplemental Fig. S6.** Bidirectional transcription changes modestly at a subset of dREG-defined (Wang et al. 2019) regulatory elements upon ZNF143 depletion.

**Supplemental Fig. S7.** ZNF143-repressed genes decrease expression in the chronic depletion background, but below our sensitivity of detection. A) Despite falling in *unchanged* and *all other genes* classes, the net response upon auxin treatment in the chronic depletion background is consistent in direction with auxin response in the rescue. Genes have a greater magnitude of response in the rescue compared to chronic depletion. B) Repressed genes in the chronic depletion background are not consistently auxin-repressed and we do not consistently observe a greater magnitude of auxin-response in the ARF-rescue. These data suggest that the changes in gene expression in the chronic depletion background may not be ZNF143-specific.

**Supplemental Fig. S8.** Auxin treatment activates Aryl Hydrocarbon Receptor (AHR) gene targets. A) Auxin treatment activates many genes compared to a DMSO control treatment. B) Auxin-activated genes are, on average, closer to AHR binding sites. C) The genes TIPARP and CYP1B1 are examples of canonical hydrocarbon-responsive genes, both of which are activated by auxin and bound by AHR. Note that the transcription start site for CYP1B1 is upstream of the gene annotation in HEK293T cells.

**Supplemental Fig. S9.** Average RNA Polymerase occupancy plots, or composite profiles, suggest that RNA Polymerase pause density is reduced in the repressed gene class with only modest differences in the other classes.

**Supplemental Fig. S10.** Individual gene analysis indicates RNA Polymerase pause density is reduced in the pause and gene body. A) Pol II pausing is consistently decreased in the auxin-repressed class. Red genes fall below an FDR threshold of 0.01. B) Each panel’s gene class was previously defined by the changes that occur over the entire gene length, which is dominated by the gene body; therefore, red genes (FDR threshold of 0.0001) are consistent with their classification. Note that the x-axis for panels A and B are not identical and only a few genes have a log_10_ mean count above 2 in the pause region for the repressed class, compared to only a few genes below a log_10_ mean count of 2 in the gene body. C) Box and whisker plots collapse the data from panels A&B above.

**Supplemental Fig. S11.** RNA Polymerase pause density is reduced in the pause region and gene body at genes that are repressed upon ZNF143 depletion. The gene body and pause region data for the repressed class from Supplemental Fig. S10 are shown side-by-side.

**Supplemental Fig. S12.** The pause index changes only modestly (log_2_ values of approximately 0) for repressed genes. A modest average decrease in pause index is also observed for the control (unchanged) gene class.
