# Supplementary figures and images for: An improved auxin-inducible degron system preserves native protein levels and enables rapid and specific protein depletion
Source: Genes Dev. 2019 Oct 1;33(19-20):1441–55. doi: 10.1101/gad.328237.119 (PMC6771385; doi:10.1101/gad.328237.119)

HEK-293T-TIR1-ZNF143-AID ARF-rescue

HEK-293T-TIR1-ZNF143-AID ARF-rescue +Auxin

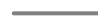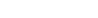

RNA Polymerase Density

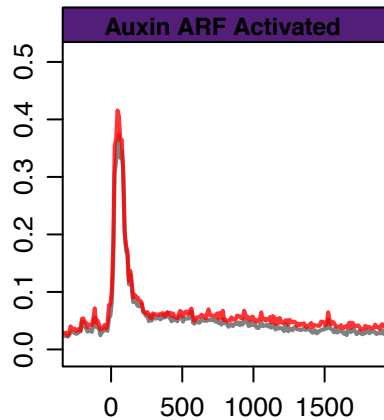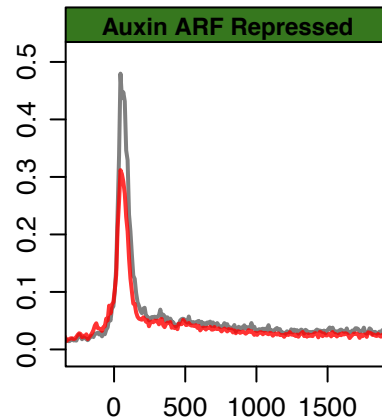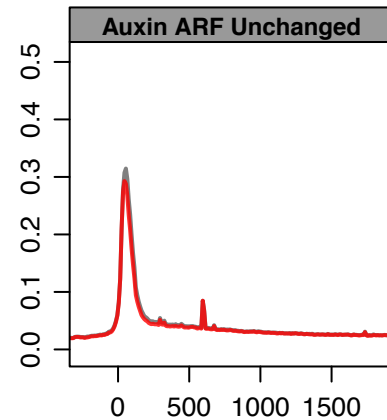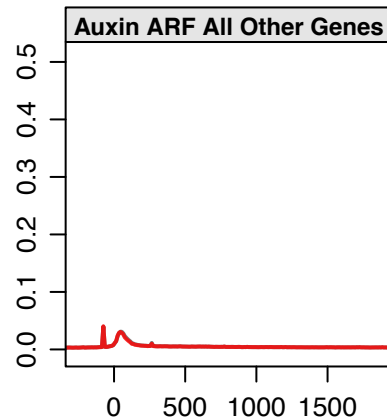

Supplement: Supplemental Material [file supp_gad.328237.119_SupplementalFigureS9.pdf]

+Auxin  $\log_2$  PRO fold change at dREG

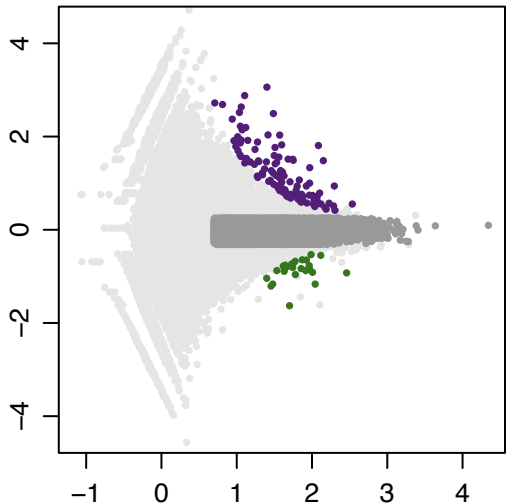

$\log_{10}$  Mean of Normalized Counts

Supplement: Supplemental Material [file supp_gad.328237.119_SupplementalFigureS6.pdf]

**A**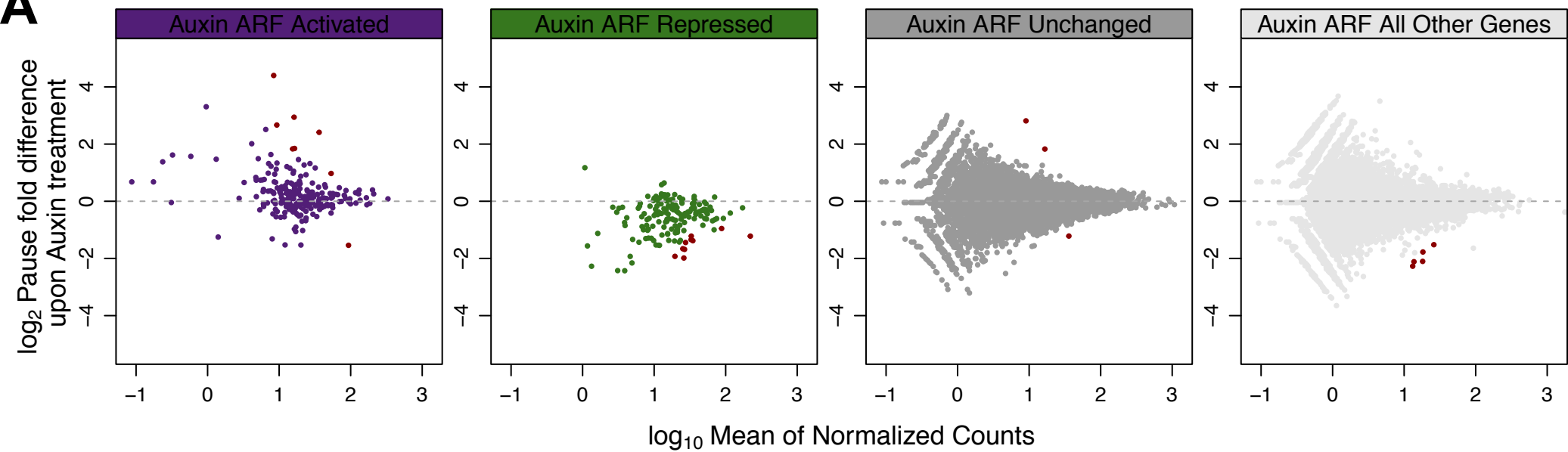**B**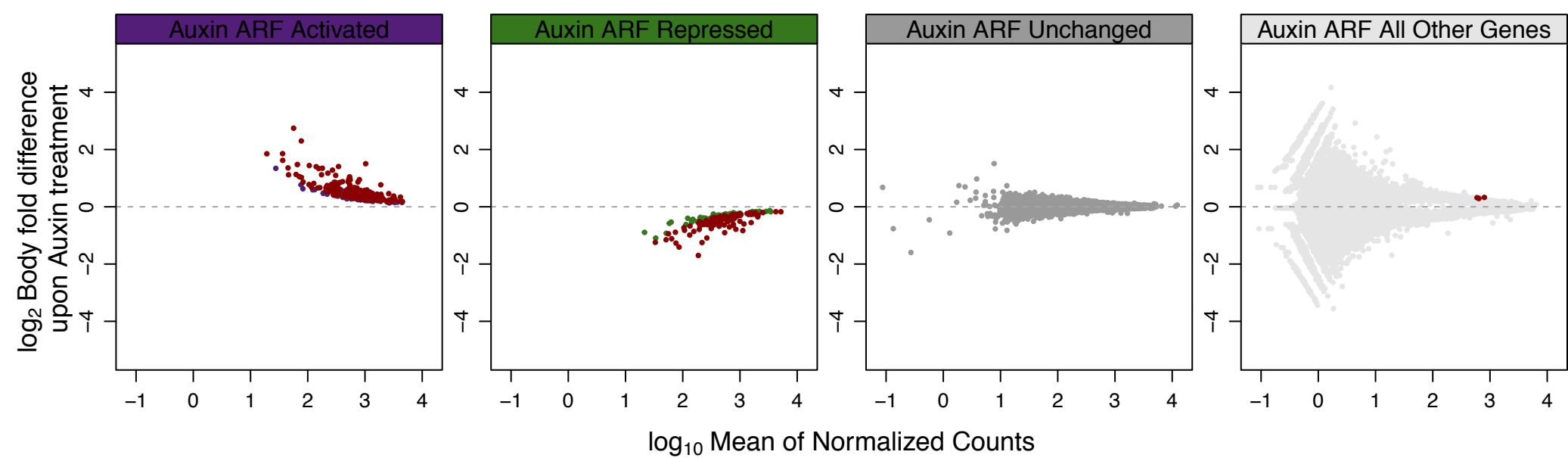**C**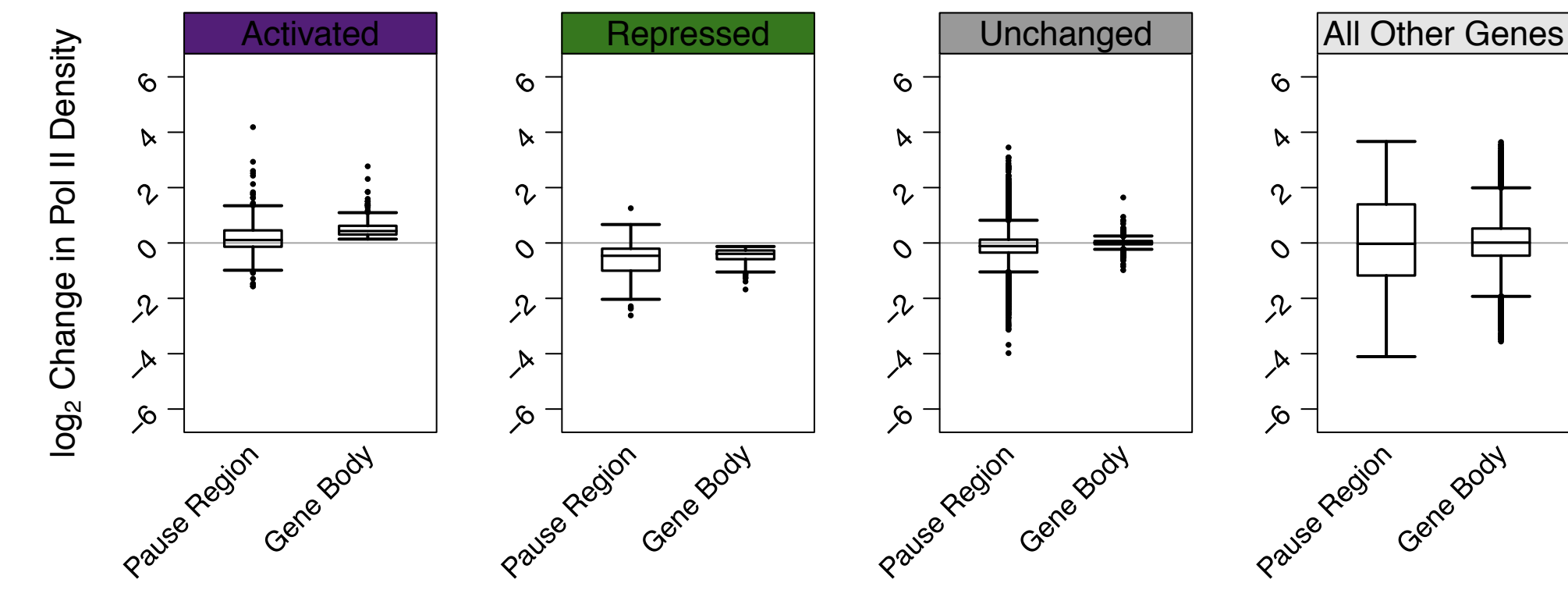

Supplement: Supplemental Material [file supp_gad.328237.119_SupplementalFigureS10.pdf]

**A**

## ARF Rescue Auxin Repressed Gene Class

$\log_2$  PRO change upon Auxin

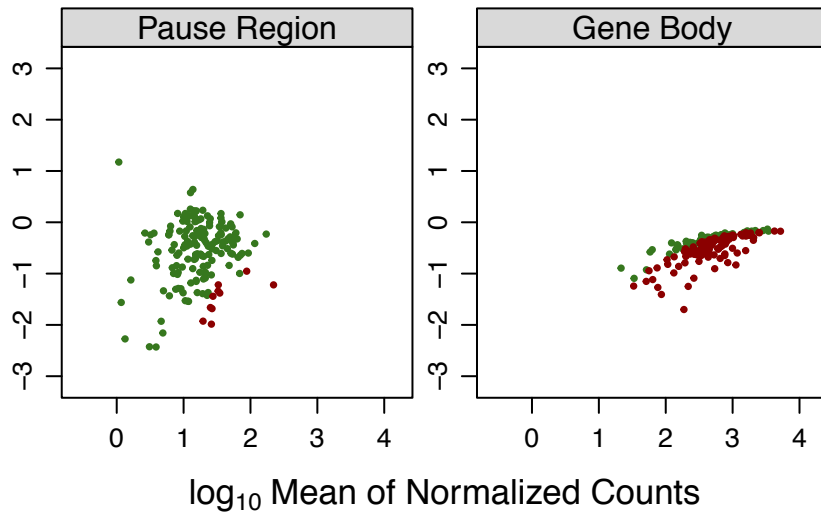**B**

$\log_2$  Change in Pol II Density

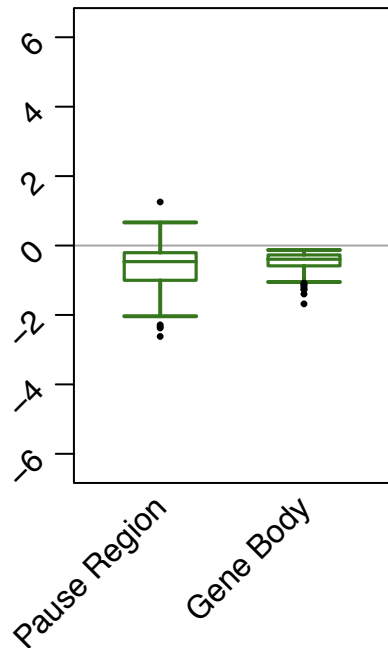

Supplement: Supplemental Material [file supp_gad.328237.119_SupplementalFigureS11.pdf]

**A**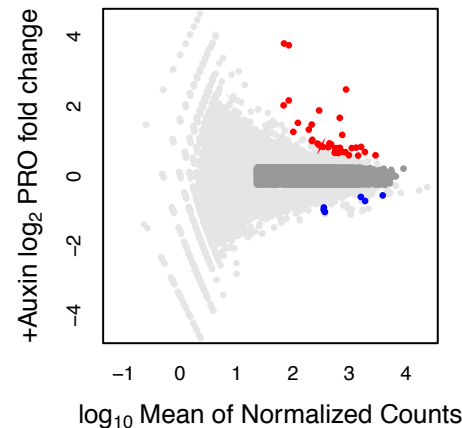**B**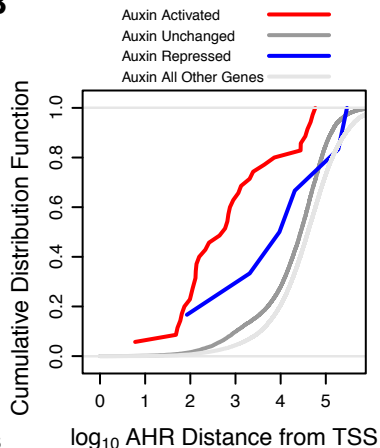**C**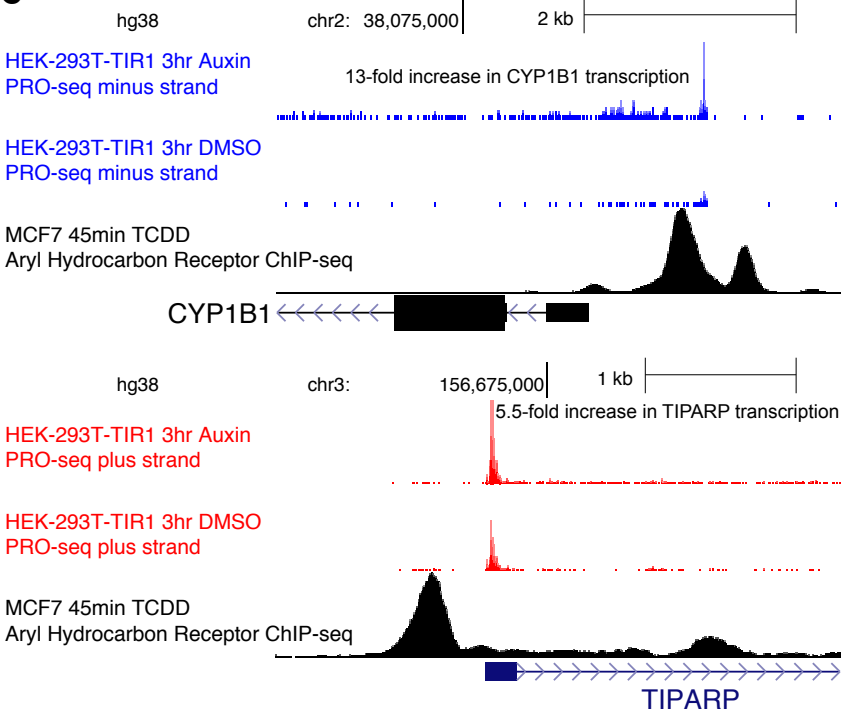

Supplement: Supplemental Material [file supp_gad.328237.119_SupplementalFigureS8.pdf]

**A**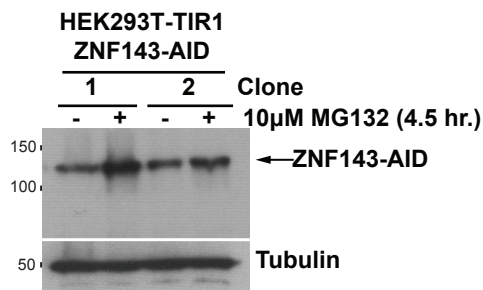**B**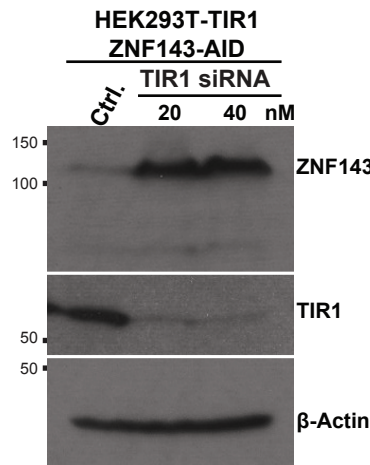**C**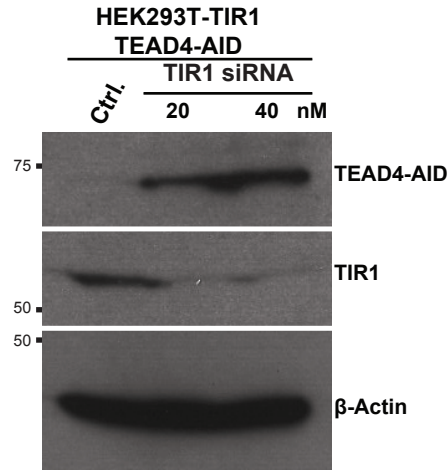**D**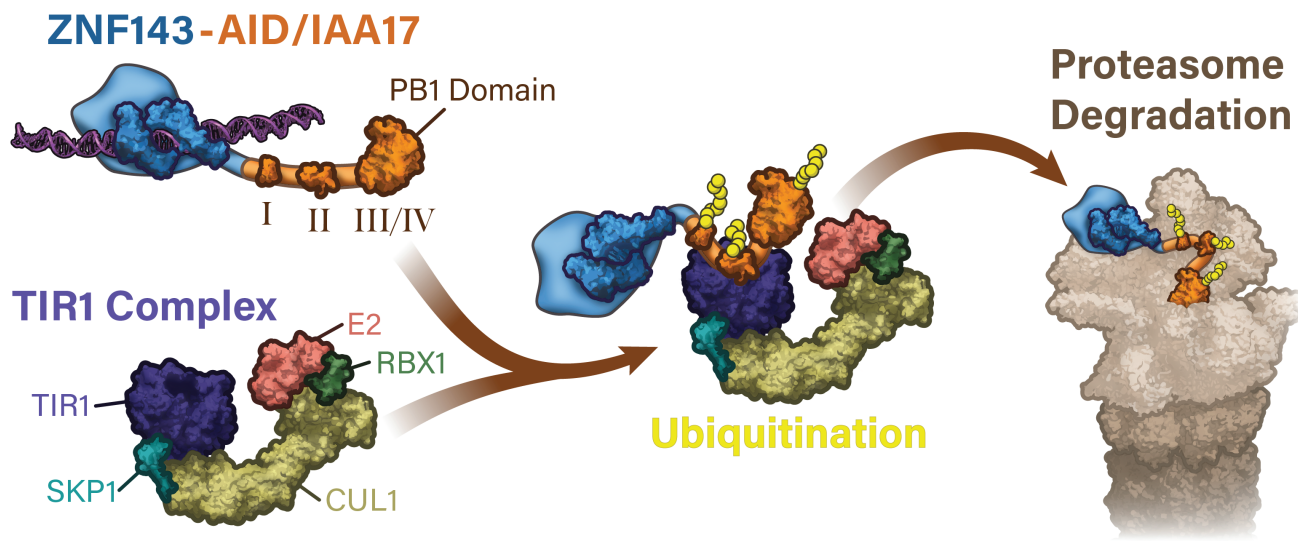

Supplement: Supplemental Material [file supp_gad.328237.119_SupplementalFigureS3.pdf]

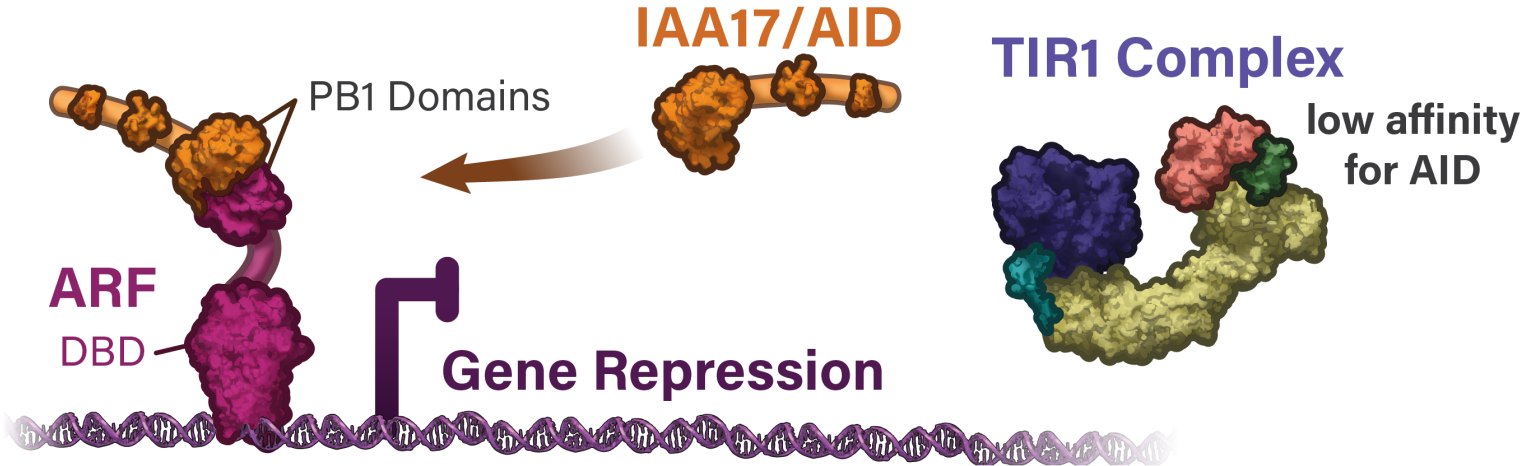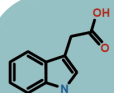

**+ Auxin**

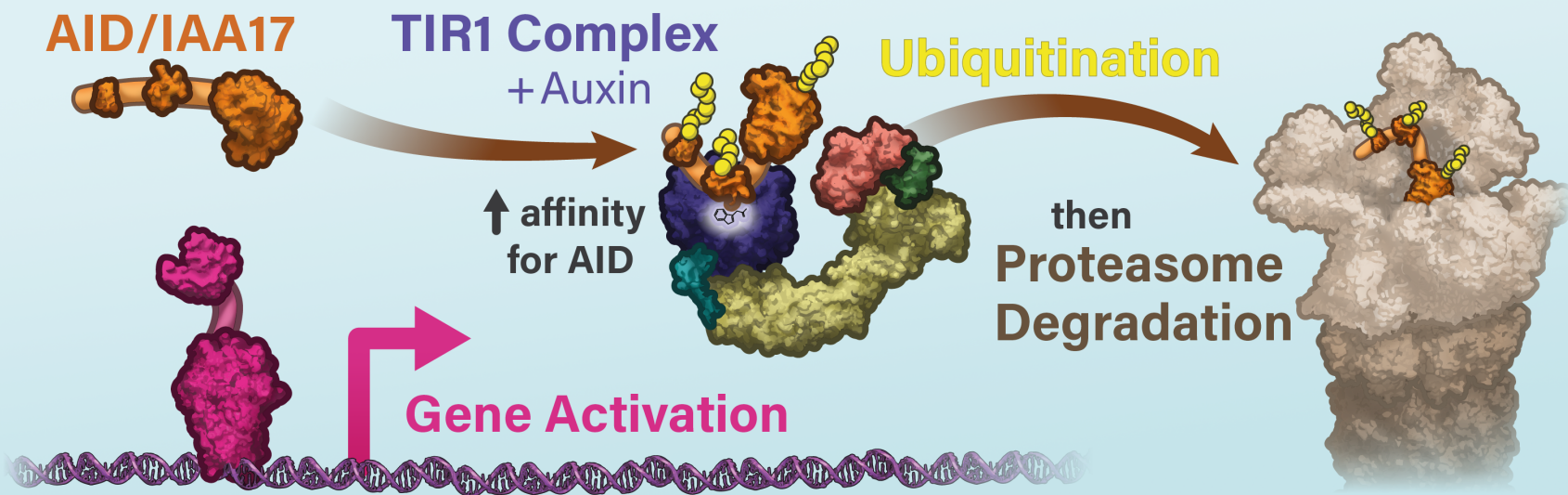

Supplement: Supplemental Material [file supp_gad.328237.119_SupplementalFigureS1.pdf]

**A**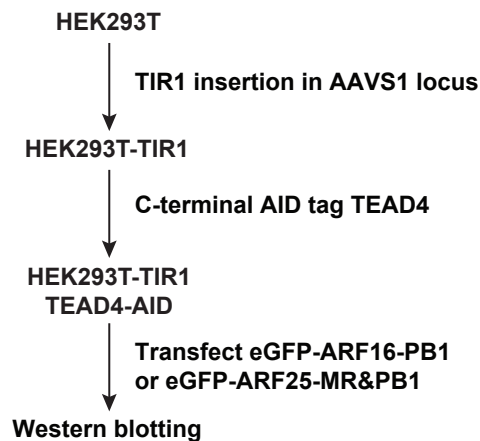**B**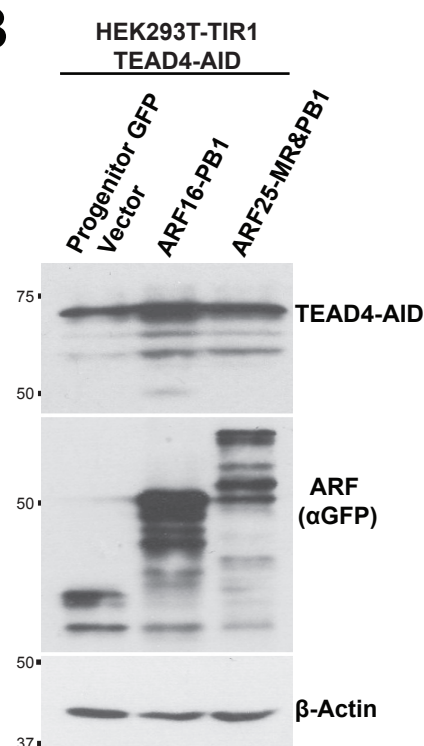**C**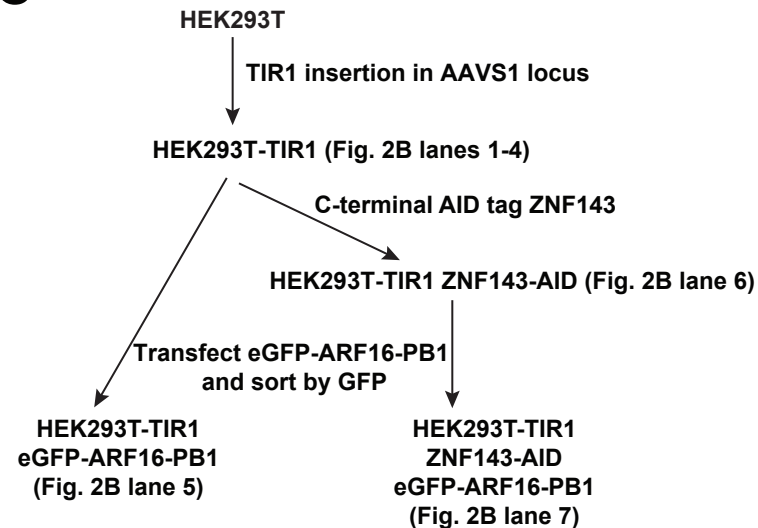**D**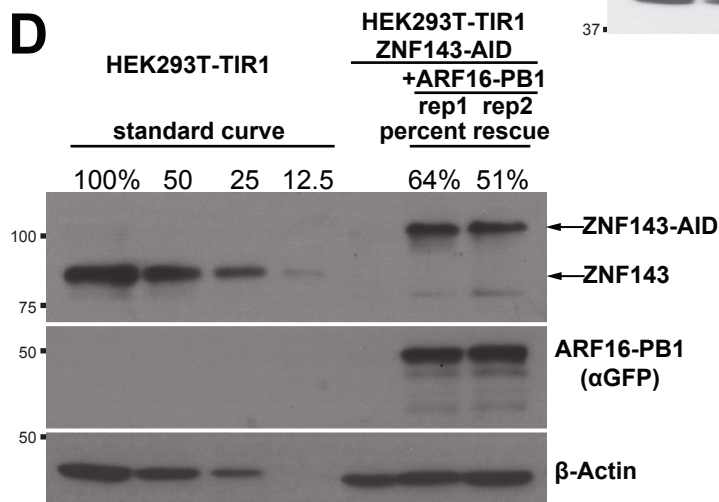**E**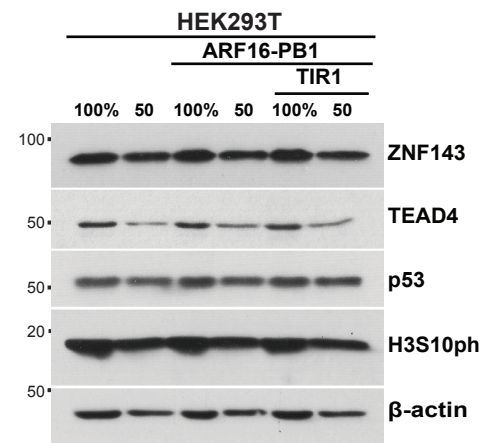

Supplement: Supplemental Material [file supp_gad.328237.119_SupplementalFigureS4.pdf]

**A**

OsTIR1 donor plasmid

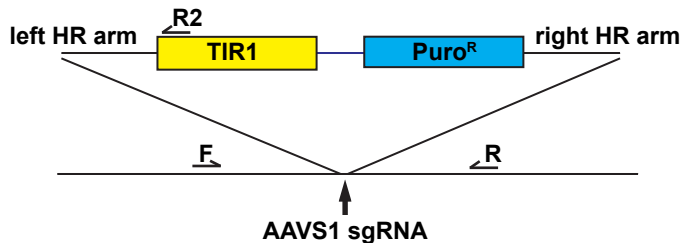**B**

genomic PCR w/ F and R

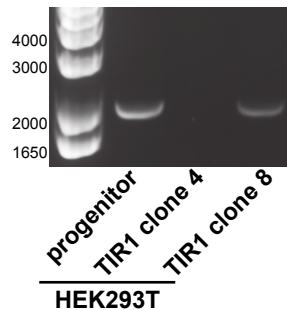**C**

genomic PCR w/ F and R2

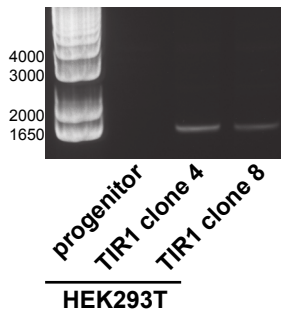**D**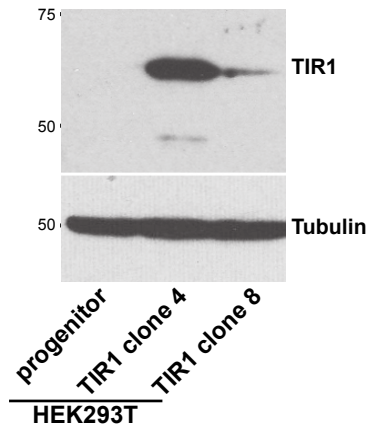

Supplement: Supplemental Material [file supp_gad.328237.119_SupplementalFigureS2.pdf]

$\log_2(\text{change in Pause Index upon Auxin})$

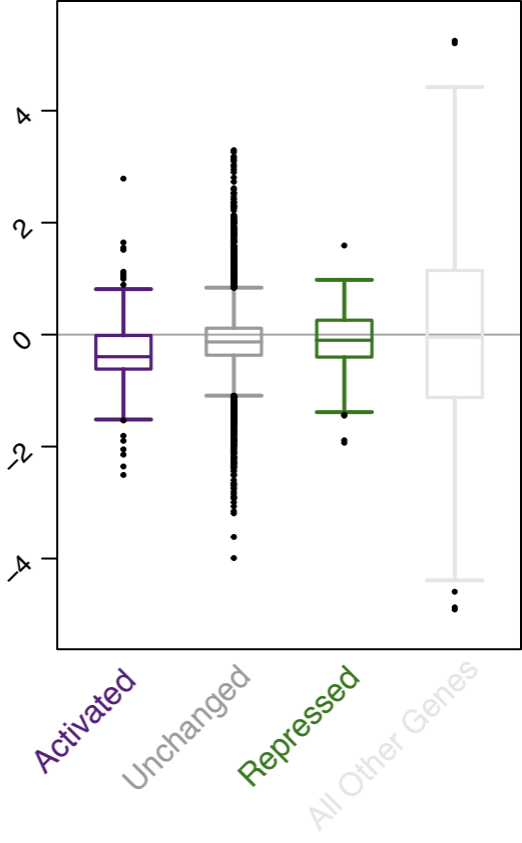

Supplement: Supplemental Material [file supp_gad.328237.119_SupplementalFigureS12.pdf]
