# Supplementary material for: An improved auxin-inducible degron system preserves native protein levels and enables rapid and specific protein depletion
Source: Genes Dev. 2019 Oct 1;33(19-20):1441–55. doi: 10.1101/gad.328237.119 (PMC6771385; doi:10.1101/gad.328237.119)
Supplement: Supplemental Material [file supp_gad.328237.119_SupplementalFigureS7.pdf]

A

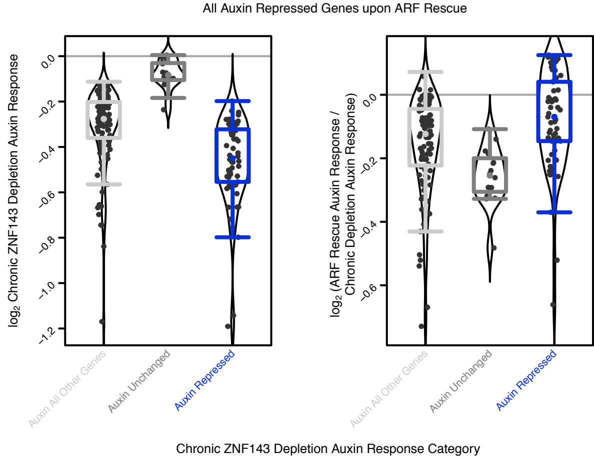
All Auxin Repressed Genes upon ARF Rescue
log2 Chronic ZNF143 Depletion Auxin Response
0.0
-0.2
-0.4
-0.6
-0.8
-1.0
-1.2
log2 (ARF Rescue Auxin Response / Chronic Depletion Auxin Response)
0.0
-0.2
-0.4
-0.6
Auxin All Other Genes
Auxin Unchanged
Auxin Repressed
Auxin All Other Genes
Auxin Unchanged
Auxin Repressed
Chronic ZNF143 Depletion Auxin Response Category

B

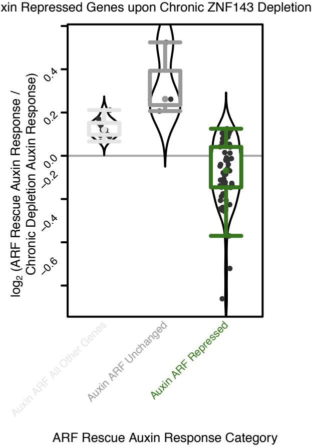
All Auxin Repressed Genes upon Chronic ZNF143 Depletion
log2 (ARF Rescue Auxin Response / Chronic Depletion Auxin Response)
0.4
0.2
0.0
-0.2
-0.4
-0.6
Auxin ARF All Other Genes
Auxin ARF Unchanged
Auxin ARF Repressed
ARF Rescue Auxin Response Category
