# Supplementary material for: An improved auxin-inducible degron system preserves native protein levels and enables rapid and specific protein depletion
Source: Genes Dev. 2019 Oct 1;33(19-20):1441–55. doi: 10.1101/gad.328237.119 (PMC6771385; doi:10.1101/gad.328237.119)
Supplement: Supplemental Material [file supp_gad.328237.119_SupplementalFigureS5.pdf]

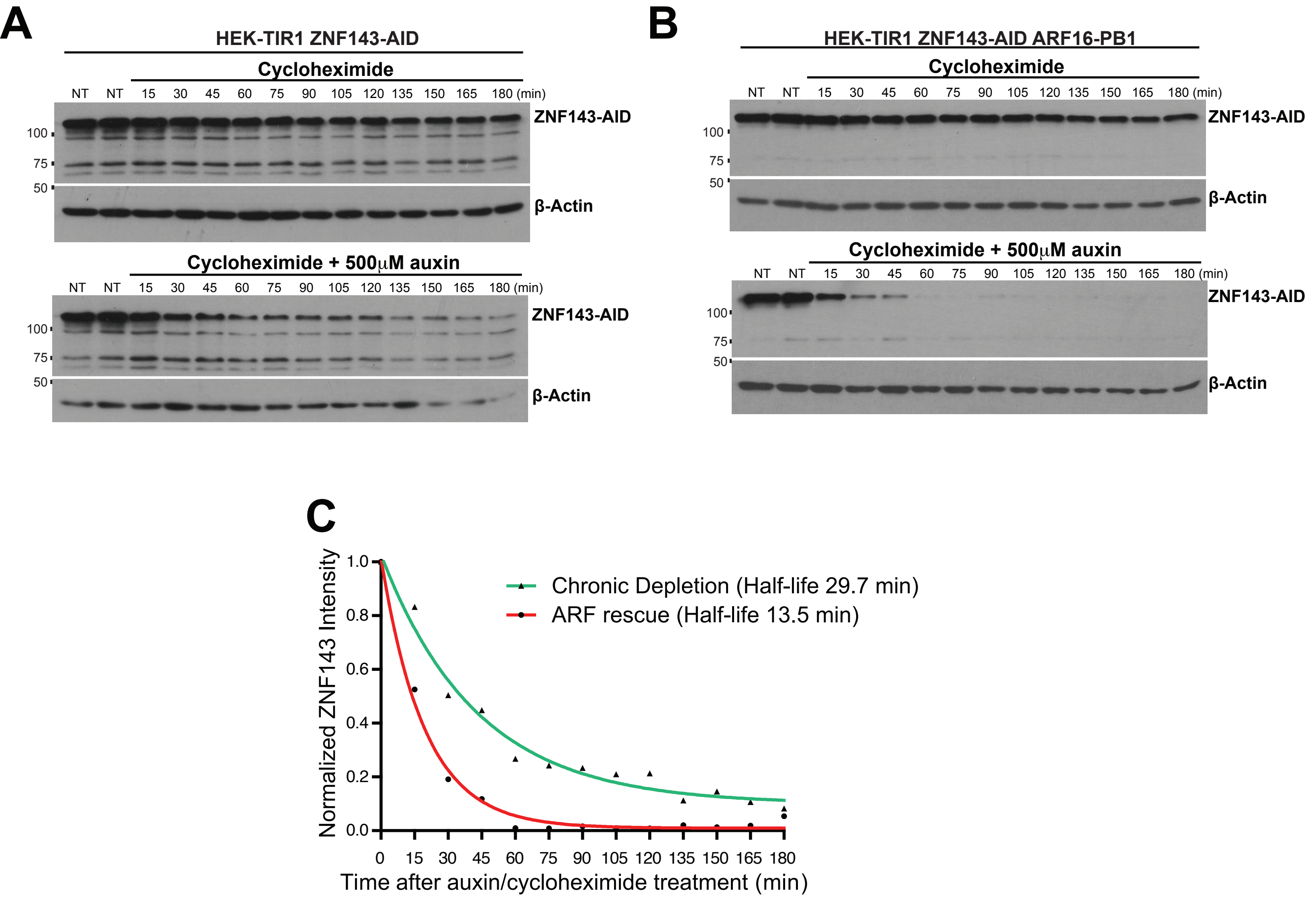
A
HEK-TIR1 ZNF143-AID
Cycloheximide
NT NT 15 30 45 60 75 90 105 120 135 150 165 180 (min)
ZNF143-AID
β-Actin
100
75
50
Cycloheximide + 500μM auxin
B
HEK-TIR1 ZNF143-AID ARF16-PB1
C
Chronic Depletion (Half-life 29.7 min)
ARF rescue (Half-life 13.5 min)
Normalized ZNF143 Intensity
Time after auxin/cycloheximide treatment (min)
1.0
0.8
0.6
0.4
0.2
0.0
0 15 30 45 60 75 90 105 120 135 150 165 180
